# Circulatory efficiency in patients with severe aortic valve stenosis before and after aortic valve replacement

**DOI:** 10.1186/s12968-020-00686-0

**Published:** 2021-03-01

**Authors:** S. Nordmeyer, C. B. Lee, L. Goubergrits, C. Knosalla, F. Berger, V. Falk, N. Ghorbani, H. Hireche-Chikaoui, M. Zhu, S. Kelle, T. Kuehne, M. Kelm

**Affiliations:** 1 Department of Congenital Heart Disease, German Heart Centre Berlin, Berlin, Germany; 2 Institute for Imaging Science and Computational Modelling in Cardiovascular Medicine, Charité-Universitätsmedizin Berlin, Augustenburger Platz 1, 13353 Berlin, Germany; 3 DZHK (German Centre for Cardiovascular Research), Partner Site Berlin, Berlin, Germany; 4 Department of Cardiothoracic and Vascular Surgery, German Heart Centre Berlin, Berlin, Germany; 5 Department of Internal Medicine and Cardiology, German Heart Centre Berlin, Berlin, Germany; 6 Department of Internal Medicine and Cardiology, Charité–Universitätsmedizin Berlin, Berlin, Germany

**Keywords:** Circulatory efficiency, Aortic valve stenosis, Heart failure, Aortic valve replacement, Remodeling, Hemodynamics

## Abstract

**Background:**

Circulatory efficiency reflects the ratio between total left ventricular work and the work required for maintaining cardiovascular circulation. The effect of severe aortic valve stenosis (AS) and aortic valve replacement (AVR) on left ventricular/circulatory mechanical power and efficiency is not yet fully understood. We aimed to quantify left ventricular (LV) efficiency in patients with severe AS before and after surgical AVR.

**Methods:**

Circulatory efficiency was computed from cardiovascular magnetic resonance (CMR) imaging derived volumetric data, echocardiographic and clinical data in patients with severe AS (n = 41) before and 4 months after AVR and in age and sex-matched healthy subjects (n = 10).

**Results:**

In patients with AS circulatory efficiency was significantly decreased compared to healthy subjects (9 ± 3% vs 12 ± 2%; p = 0.004). There were significant negative correlations between circulatory efficiency and LV myocardial mass (r = − 0.591, p < 0.001), myocardial fibrosis volume (r = − 0.427, p = 0.015), end systolic volume (r = − 0.609, p < 0.001) and NT-proBNP (r = − 0.444, p = 0.009) and significant positive correlation between circulatory efficiency and LV ejection fraction (r = 0.704, p < 0.001). After AVR, circulatory efficiency increased significantly in the total cohort (9 ± 3 vs 13 ± 5%; p < 0.001). However, in 10/41 (24%) patients, circulatory efficiency remained below 10% after AVR and, thus, did not restore to normal values. These patients also showed less reduction in myocardial fibrosis volume compared to patients with restored circulatory efficiency after AVR.

**Conclusion:**

In our cohort, circulatory efficiency is reduced in patients with severe AS. In 76% of cases, AVR leads to normalization of circulatory efficiency. However, in 24% of patients, circulatory efficiency remained below normal values even after successful AVR. In these patients also less regression of myocardial fibrosis volume was seen.

*Trial Registration* clinicaltrials.gov NCT03172338, June 1, 2017, retrospectively registered.

## Introduction

Aortic valve stenosis (AS) is a frequent heart valve disease worldwide that exposes the left ventricle (LV) to chronic pressure overload [1–3]. This triggers a complex cascade of LV remodeling processes leading to hypertrophy and fibrosis [1, 2] and if treatment is performed too late regression of these LV remodeling processes is reduced and morbidity as well as mortality increase [4, 5].

Circulatory efficiency reflects the ratio between total LV work and the work required for maintaining cardiovascular circulation [6–9]. The approach might contribute to the understanding of potential regenerative processes in the pressure overloaded heart [10, 11]. In fact, only part of the LV work is directly used to maintain blood flow in the cardiovascular circulation in AS patients, the rest is needed to build up the pressure to overcome the resistance across the aortic valve and parts dissipate as heat [8].

Increases in LV pressure and myocardial hypertrophy can contribute to a reduction of cardiac efficiency, whereas small ventricles with normal LV ejection fraction (LVEF) show higher cardiac efficiency. Accordingly, the concept of cardiac efficiency has been analyzed in some initial studies of arterial hypertension, heart failure and valve disease [7, 11–14]. Güclu et al. have reported in a positron emission tomography (PET) cardiovascular magnetic resonance (CMR) study that efficiency is a determinant of functional improvement after aortic valve replacement (AVR) in patients with AS [13, 14]. However, the study was performed only on a small group of 10 patients and therefore more clinical data is warranted. The acquisition of clinical data, however, can be technically challenging and methods that were used in the past were often invasive or associated with ionizing radiation – thus limiting their clinical use.

A reduced surrogate marker of circulatory efficiency in patients with different stages of AS has been found using a recent noninvasive and radiation-free CMR method [15]. In the present study, we aimed to apply this novel noninvasive method to assess surrogate markers of circulatory efficiency and power in a cohort of 41 patients with severe AS before and after surgical AVR and in 10 age and sex-matched controls.

## Methods

### Study design and data acquisition

A total of 41 patients with severe AS (according to current diagnostic guidelines [16]) were included into the study (Table 1). Exclusion criteria were the presence of moderate to severe aortic regurgitation (AR), mitral, pulmonary or tricuspid valve disease [17], the presence of coronary artery disease and general contraindications to CMR.

**Table 1:** General demographic and clinical data; mean ± SD and n (%)

Subjects	AS patients (n = 41)	Healthy controls (n = 10)	p-value
Age (years)	67 ± 9	62 ± 10	0.131
Male gender, n (%)	21 (51%)	5 (50%)	0.945
Body mass index (kg/m^2^)	28 ± 4.2	25 ± 3.6	0.029
BSA (m^2^)	2 ± 0.24	2 ± 0.2	0.169
Duration between AVR and Visit 2 (d)	125 ± 38	–	–
Prothesis data			
Prosthesis size [mm]	23 ± 2	–	–
Biological prosthesis, n (%)	39 (95%)	–	–
Prosthesis type Medtronic biological, n (%)	1 (2%)	–	–
Prosthesis type Medtronic mechanical, n (%)	1 (2%)	–	–
Prosthesis type Edwards, n (%)	9 (22%)	–	–
Prosthesis type St Jude Medical Regent, n (%)	1 (2%)	–	–
Prosthesis type Trifecta, n (%)	21 (51%)	–	–
Prosthesis type CE Perimount Magna Ease, n (%)	8 (20%)	–	–
Risk factors			
Bicuspid aortic valve, n(%)	31 (76%)	0 (0%)	< 0.001
Dyslipidaemia, n(%)	20 (49%)	4 (40%)	0.618
Diabetes mellitus, n(%)	4 (10%)	1 (10%)	0.981
CCS III-VI, n(%)	3 (7%)	0 (0%)	0.378
NYHA III-VI, n(%)	18 (35%)	0 (0%)	0.009
Arterial hypertension, n(%)	29 (71%)	4 (40%)	0.068
Medical treatment			
Betablocker, n (%)	17 (42%)	3 (30%)	0.506
Calcium antagonist, n (%)	4 (10%)	1 (10%)	0.981
Diuretics, n (%)	11 (27%)	2 (20%)	0.657
ARB, n (%)	9 (22%)	2 (20%)	0.893
ACE-I, n (%)	11 (29%)	0 (0%)	0.064

*BSA* body surface area, *RF* regurgitation fraction, *CCS* Canadian Cardiovascular Society, *NYHA* New York Heart Association, *ARB* angiotensin receptor blocker, *ACE-I* angiotensin converting encyme inhibitor

All patients underwent cuff-based blood pressure measurements, blood collection, clinical, echocardiographic and CMR examination before and 4 (± 38 days) months after surgical AVR. The mean and maximum pressure gradient across the aortic valve was measured using Doppler echocardiography (5-chamber-view). Mitral regurgitation was quantified using standard echocardiography. 10 age- and sex-matched controls (Table 1) underwent the same pre-operative study protocol and were compared to the AS patients. The study protocol was in agreement with the principles outlined in the Declaration of Helsinki and was approved by the Medical Ethics Review Committee. All patients gave written informed consent prior to inclusion.

### Cardiovascular magnetic resonance imaging and post-processing

All CMR examinations were performed using a whole-body 1.5 T CMR system (Achieva R 3.2.2.0, Philips Healthcare, Best, The Netherlands) using a five-element cardiac phased-array coil. Gapless balanced Turbo Field Echo (bTFE) cine 2-dimensional short axis sequences were obtained using a previously applied CMR protocol [8] for LV volumetric and anatomical measurements. Analysis was performed using View Forum (R6.3V1L7 SP1; Philips Healthcare). LV epicardial and endocardial borders were manually drawn in every segment in diastole and systole to acquire automatically LV volumetric and anatomical data (LV mass (LVM), myocardial volume, end-systolic volume (ESV) and end-diastolic volume (EDV)). End-systolic mean myocardial wall thickness ${S}_{wall}$ and mean radius of the blood pool ${R}_{BP}$ were calculated considering the LV a cylinder:

$${R}_{BP} ={\left(\frac{{V}_{BP}}{{n}_{cine}*{h}_{cine}*\pi }\right)}^{1/2}$$

$${S}_{wall} ={\left(\frac{{{V}_{wall}+ V}_{BP}}{{n}_{cine}*{h}_{cine}*\pi }\right)}^{1/2}-{R}_{BP}$$

where ${n}_{cine}$ = number of 2D Cine CMR slices used for the LV volumetric measurements, ${h}_{cine}$ = Cine slice thickness (usually 7 mm), ${V}_{BP}$ = blood pool volume and ${V}_{wall}$ = myocardial wall volume.

4-dimensional and 2-dimensional velocity encoded (VENC) CMR was obtained using a previously described CMR protocol [18]. 4-D VENC CMR sequences were used to quantify blood flow across the aortic and mitral valve and the ascending aorta in order to measure auxobaric contraction time t_ABC_, isovolumetric contraction time t_IVC_ and the aortic pressure gradient. 4D data were analyzed using GT Flow program (version 2.0.10, Gyrotools, Zurich, Switzerland). Total systolic contraction time t_CS_ is the sum of t_ABC_ and t_IVC_. The temporal solution was 25 timesteps for 4D flow measurements and 30 timesteps for 2D flow measurements. We could show that 4D flow measurements with 25 timesteps are a feasible alternative to flow measurements with higher temporal solution in a prior study [15].

### Global Longitudinal Strain (GLS) Feature-tracking (CMR-FT)

CMR-FT based strain analyses were performed using commercially available software provided by Medis (QStrain, Version 2.1.12.2, Medis Medical Imaging Systems, Leiden, Nethrlands). FT was performed in the end-diastole and end-systole cardiac phases, at the endo- and epicardial borders. Global longitudinal strain (GLS) was assessed by averaging the peak systolic strain values of 17 segments extracted from three long axis  images (2-, 3- and 4-long axis CV), while global circumferencial strain (GCS) was acquired from three short axis images (basal, midventricular and apical level) using a 16-segment model.

### Circulatory power and efficiency (Fig. 1)

**Fig. 1:**
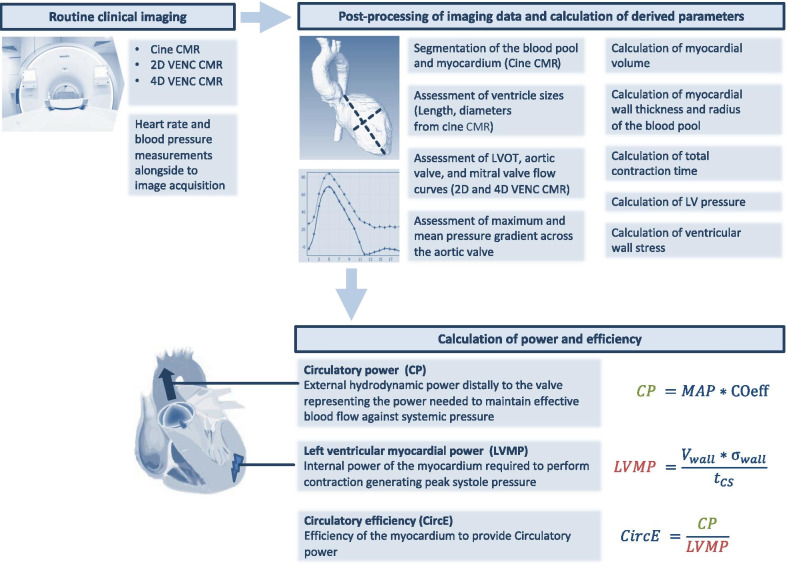
Illustration of the processing pipeline and the calculation of power and efficiency. After the initial acquisition of routine cine CMR and 2D/4D velocity encoded (VENC) CMR as well as heart rate and blood pressure measurements, image segmentation and the assessment of functional parameters is performed. These informations are required to calculate the mechanical circulatory power (CP), the left ventricular (LV) myocardial power (LVMP) and the resulting efficiency

Power is the rate of transferring or converting energy per unit time. The ratio of the power needed to pump a given blood volume against a given afterload (circulatory power, CP) to the power used by the heart to perform one heartbeat (Left ventricular myocardial power, LVMP) is described as circulatory efficiency (CircE). LVMP was defined as the surrogate power of the LV to perform one heartbeat since the applied method is an estimation [15, 19]:

$$LVMP =\frac{{V}_{wall}*{\upsigma }_{wall}}{{t}_{CS}}$$

V_wall_ = myocardial wall volume, σ_wall_ = wall stress, t_CS_ = LV systolic contraction time.

Wall stress was calculated using a simplified approach of the law of Laplace:

$${\upsigma }_{wall} ={P}_{sys}* \frac{{R}_{BP}}{2*{S}_{wall}}$$

P_SYS_ = LV peak systolic pressure, R_BP_ = mean radius of the blood pool, S_wall_ = mean myocardial wall thickness. S_wall_ and R_BP_ during systole were averaged from LV segmentations considering the LV as a cylindrical geometry for correction of potential regional differences. P_sys_ = sum of the systolic blood pressure measured at the right arm and the maximum pressure gradient across the aortic valve. LVMP was indexed to body surface area (BSA).

**Circulatory power (CP)** is defined as the hydrodynamic power distally to the valve representing the power needed to maintain effective blood flow against systemic vascular resistance (afterload).

$$CP =MAP*\mathrm{COeff}$$

MAP = mean arterial pressure, CO_eff_ = effective cardiac output. The dimension of CO_eff_ is L/min. CO_eff_ is the product of heart rate and SV_eff_. SV_eff_ = (EDV-ESV) * (1-regurgitation fraction).

**Circulatory efficiency (CircE)** is the ratio between CP and LVMP.

$$CircE =\frac{CP}{LVMP}$$

### Calculation of diffuse myocardial fibrosis

Calculation of extracellular volume (ECV) was done using a prior described method [20, 21]:

$$ECV=\left(1-hematocrit\right)* \frac{(1/T myo post)-(1/T myo pre)}{(1/T blood post)-(1/T blood pre)}$$

myo = LV midwall myocardial T1 value, blood = LV blood pool T1 value, and pre and post refers to the measurement before and after contrast administration. Absolute ECV was calculated using the following equation: aECV = LV myocardial volume*ECV. LV myocardial volume = LV mass/1.05, where 1.05 is the myocardial density given in g/ml.

### Reproducibility of power and efficiency measurements

As stated in a prior study [15], parameters of circulatory power and efficiency are combined parameters calculated from CMR acquired LV volumetric and 2D/4D blood flow measurements. Several previous studies have shown good reproducibility and accuracy of CMR acquired LV volumetric and 2D/4D blood flow measurements [18, 22–24].

### Statistics

Data are presented as mean ± SD unless stated otherwise. Shapiro–Wilk test was used for normality testing. A paired two-tailed Student’s t-test or Wilcoxon test was performed where appropriate to investigate differences between pre- and post-operative measurements. Unpaired two-tailed Student´s t-test or Mann–Whitney-U Test was performed to investigate differences between disease groups and controls as appropriate. Pearson´s chi-square test was performed to investigate differences in unpaired categorical variables. McNemar test was used to test for differences in paired categorical data before and after an intervention. A linear regression model was used to identify the relationship between variables. The significance level was set at 0.05. Data were analyzed using SPSS (version 25, Statistical Package for the Social Sciences, International Business Machines, Inc., Armonk, New York, USA) and Stata (Version 15.1, StataCorp, College Station, Texas, USA).

## Results

### Clinical effects of AVR

**Fig. 2:**
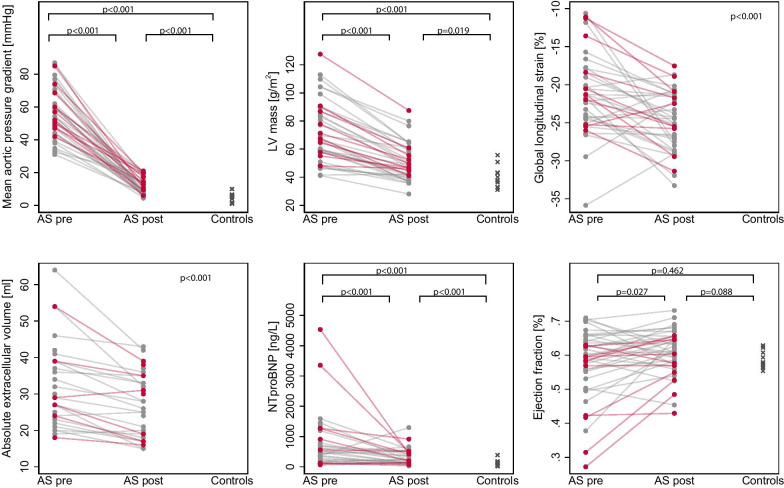
Measurements in patients with aortic stenosis (AS) before and after aortic valve replacement (AVR) and in controls. First row (left, middle, right): Mean aortic pressure gradient, left ventricular (LV) mass, global longitudinal strain (GLS), Second row (left, middle, right): absolute extra cellular volume, NTpro-BNP and LV ejection fraction

Table 1 summarizes demographic and clinical baseline characteristics. Table 2 shows CMR, laboratory and clinical parameters in patients and controls. In patients with AS mean aortic pressure gradient decreased and New York Heart Association (NYHA) classification improved after AVR. Furthermore, there was a significant reduction in hypertrophy (myocardial muscle mass), fibrosis (aECV) and NT-proBNP and a significant increase in GLS (Fig. 2).

**Table 2:** CMR, metabolic and clinical parameters before and after aortic valve replacement (AVR) and in healthy controls

Parameters	Pre-AVR (n = 41)	Post-AVR (n = 41)	Healthy controls (n = 10)	P value pre-AVR vs post-AVR	P value pre-AVR vs healthy controls	P values post-AVR vs healthy controls
CMR parameters						
Myocardial mass/BSA [mg/m^2^]	72 ± 21	50 ± 13	40 ± 8	< 0.001	< 0.001	0.019
LVESD [mm]	34 ± 6	32 ± 4	34 ± 2	< 0.001	0.943	0.035
Myocardial wall thickness end systolic [mm]	11 ± 2	10 ± 2	8 ± 1	< 0.001	< 0.001	0.001
LVEDV/BSA [ml/m^2^]	93 ± 22	77 ± 13	82 ± 9	< 0.001	0.117	0.169
LVESV/BSA [ml/m^2^]	41 ± 18	31 ± 9	34 ± 4	< 0.001	0.393	0.064
LVEF [%]	58 ± 10	61 ± 7	59 ± 3	0.027	0.462	0.088
LV mass-volume-index [g/ml]	0.8 ± 0.2	0.7 ± 0.2	0.5 ± 0.1	0.001	< 0.001	< 0.001
RF [%]	9 ± 6	7 ± 5	3 ± 2	0.115	0.004	0.005
Heart rate [bpm]	67 ± 11	70 ± 12	62 ± 10	0.189	0.302	0.104
CO_total [l/min]	6.8 ± 1.9	6.3 ± 1.7	5.4 ± 1.2	0.048	0.041	0.184
CI [(l/min)/m^2^]	3.5 ± 0.8	3.2 ± 0.7	2.9 ± 0.4	0.056	0.039	0.265
SV_total [ml]	79 ± 20	76 ± 15	74 ± 19	0.396	0.440	0.440
ECV midwall [%]	23 ± 2	27 ± 3		< 0.001	–	–
aECV midwall [ml]	32 ± 11	27 ± 9		< 0.001	–	–
aECV/BSA midwall [ml/m^2^]	16 ± 5	14 ± 4		< 0.001	–	–
GLS [%]	− 21.2 ± 5	− 24.8 ± 4		< 0.001	–	–
Late gadolinium enhancement, n (%)	5 (16%)	8 (23%)	–	1.000	–	–
Mitral regurgitation II-IV, n (%)	0 (0%)	1 (2%)	0 (0%)	1.000	–	0.618
Heart power parameters						
LV myocardial power [W/m^2^]	8 ± 3	5 ± 2	5 ± 0.8	< 0.001	< 0.001	0.924
Circulatory power [W]	1.3 ± 0.4	1.3 ± 0.4	1.1 ± 0.3	0.462	0.097	0.462
Circulatory efficiency [%]	9.3 ± 3	13.3 ± 5	12.3 ± 2	< 0.001	0.004	0.112
Circulatory efficiency improvement [%]	–	4.1 ± 5.7	–	–	–	–
Metabolic parameters						
NT-pro-BNP [ng/l]	770 ± 926	334 ± 261	113 ± 113	< 0.001	< 0.001	0.001
Hemoglobin [g/dl]	14.2 ± 1.3	13.5 ± 1.3	13.9 ± 0.8	0.011	0.607	0.184
Hematocrit [%]	42 ± 4	42 ± 4	41 ± 3	0.609	0.511	0.497
Clinical parameters						
NYHA III-IV, n(%)	18 (35%)	3 (7%)	0 (0%)	< 0.001	0.009	0.378
Systolic blood pressure [mmHg]	137 ± 17	138 ± 19	136 ± 15	0.650	0.704	0.877
Diastolic blood pressure [mmHg]	75 ± 10	75 ± 9	74 ± 9	0.872	0.868	0.831
Mean arterial pressure [mmHg]	96 ± 11	96 ± 11	95 ± 9	0.948	0.803	0.822
Mean aortic pressure gradient [mmHg]	56 ± 15	11 ± 5	5 ± 3	< 0.001	< 0.001	< 0.001
Maximal aortic pressure gradient [mmHg]	83 ± 22	19 ± 7	9.5 ± 5	< 0.001	< 0.001	< 0.001
Pulse pressure [mmHg]	63 ± 16	63 ± 16	62 ± 13	0.906	0.972	0.905
6-min walk distance [m]		518 ± 84	538 ± 111	–	–	0.646

LVESD, left ventricular end systolic diameter; EDV, end diastolic volume; ESV, end systolic volume; EF, ejection fraction; RF, regurgitation fraction; HR, heart rate; CO, cardiac output; ECV, extracellular volume; aECV, absolute extracellular volume; GLS, global longitudinal strain; NT-pro-BNP, N terminales pro brain natriuretic peptide; NYHA, New York Heart Association

**Table 3:** General demographic and clinical data in patients with and without restored circulatory efficiency (CircE)  after AVR

	not restored CircE (n = 10)	restored CircE (n = 31)	p-value
Age (years)	67 ± 7.8	66 ± 9.7	0.917
Male gender, n (%)	7 (70%)	14 (45%)	0.172
Body mass index (kg/m^2^)	28 ± 5.1	28 ± 4.0	0.580
BSA (m^2^)	2 ± 0.3	2 ± 0.2	0.580
Prothesis size [mm]	24 ± 2	23 ± 2	0.247
Biological prosthesis, n (%)	10 (100%)	29 (94%)	0.410
Prosthesis type Medtronic biological, n (%)	1 (10%)	0 (0%)	0.075
Prosthesis type Medtronic mechanical, n (%)	0 (0%)	1 (3%)	0.565
Prosthesis type Edwards, n (%)	2 (20%)	7 (23%)	0.864
Prosthesis type SJM regent, n (%)	0 (0%)	1 (3%)	0.565
Prosthesis type Trifecta, n (%)	5 (50%)	16 (52%)	0.929
Prosthesis type CE Perimount Magna Ease, n (%)	2 (20%)	6 (19%)	0.964
Risk factors			
Bicuspid aortic valve, n (%)	8 (80%)	23 (74%)	0.710
Dyslipidaemia, n (%)	7 (70%)	13 (42%)	0.123
Diabetes mellitus, n (%)	2 (20%)	2 (6%)	0.209
Arterial hypertension, n (%)	8 (80%)	21 (68%)	0.459
CCS III-VI, n (%)	1 (10%)	2 (6%)	0.708
NYHA III-IV, n [%]	5 (50%)	13 (42%)	0.655
Medical Treatment			
Betablocker, n (%)	4 (40%)	13 (42%)	0.914
Calcium antagonist, n (%)	1 (10%)	3 (10%)	0.976
Diuretics, n (%)	3 (30%)	8 (26%)	0.795
ARB, n (%)	3 (30%)	6 (19%)	0.479
ACE-I, n (%)	4 (40%)	7 (23%)	0.280

*BSA* body surface area, *CCS* Canadian Cardiovascular Society, *NYHA* New York Heart Association, *ARB* angiotensin receptor blocker, *ACE-I* Angiotensin converting enzyme inhibitor

**Table 4:** Pre- and Post-AVR parameters in patients with and without restored CircE post-AVR

	Pre-AVR not restored CircE (n = 10)	Post-AVR not restored CircE (n = 10)	Pre-AVR restored CircE (n = 31)	Post-AVR restored CircE (n = 31)
Power and efficiency				
LV myocardial power/BSA [W/m^2^]	9.5 ± 3.3	6.9 ± 2.0*^+^	7.5 ± 2.6	4.7 ± 1.5*
Circulatory power [W]	1.3 ± 0.4	1.1 ± 0.4	1.3 ± 0.4	1.3 ± 0.4
Circulatory efficiency [%]	7.8 ± 3.2	8.5 ± 0.9^+^	9.7 ± 3.2	14.9 ± 5.1*
Circulatory efficiency improvement [%]	–	0.6 ± 2.8^+^	–	5.2 ± 6.0
Conventional parameters of cardiac vascular function				
Myocardial mass/BSA [mg/m^2^]	75 ± 23	53 ± 13*	71 ± 21	49 ± 13*
Myocardial wall thickness end systolic [mm]	11 ± 2	10 ± 1*	11 ± 2	10 ± 2*
LVEDV/BSA [ml/m^2^]	99 ± 24	78 ± 14*	92 ± 21	77 ± 13*
LVESV/BSA [ml/m^2^]	52 ± 27	34 ± 11*	37 ± 13	30 ± 8*
Mass-volume-index [g/ml]	0.8 ± 0.1	0.7 ± 0.1*	0.8 ± 0.2	0.7 ± 0.2*
LVESD [mm]	38 ± 7	33 ± 4*	33 ± 5	31 ± 4*
Left atrium [cm^2^]	50 ± 15	43 ± 11	48 ± 17	47 ± 15
LVP [mmHg]	224 ± 21	164 ± 23*	219 ± 26	154 ± 20*
Heart rate [bpm]	68 ± 11	67 ± 10	66 ± 11	70 ± 12
CO_total [l/min]	6.4 ± 1.4	5.8 ± 1.2	7.0 ± 2.0	6.5 ± 1.8
CI [(l/min)/m^2^]	3.2 ± 0.7	3.0 ± 0.5	3.6 ± 0.8	3.3 ± 0.8
SV_total [ml]	73 ± 18	73 ± 14	81 ± 20	77 ± 15
ECV midwall [%]	24 ± 2	28 ± 5	23 ± 2	27 ± 3*
aECV midwall [ml]	32 ± 13	28 ± 10	32 ± 11	26 ± 8*
aECV/BSA midwall [ml/m^2^]	17 ± 7	15 ± 5	16 ± 5	13 ± 3*
GLS [%]	− 20 ± 6	− 24 ± 4*	− 22 ± 5	− 25 ± 4*
LVEF [%]	50 ± 13^+^	57 ± 7*	60 ± 8	62 ± 6
LGE, n(%)	2 (20%)	2 (20%)	3 (10%)	6 (19%)
Metabolic parameters				
NT-pro-BNP [ng/l]	1356 ± 1570	346 ± 260*	559 ± 427	329 ± 266*
Hemoglobin [g/dl]	14.7 ± 1.0	13.3 ± 1.0*	14.0 ± 1.4	13.6 ± 1.3*
Hematocrit [%]	43 ± 3	42.4 ± 3.1	41 ± 4	41.9 ± 3.8
Clinical parameters				
Mean aortic pressure gradient [mmHg]	58 ± 14	15 ± 5*^+^	56 ± 16	10 ± 5*
Systolic blood pressure [mmHg]	140 ± 12	142 ± 21	137 ± 19	136 ± 19
Diastolic blood pressure [mmHg]	80 ± 6^+^	74 ± 8	73 ± 11	75 ± 9
Pulse pressure [mmHg]	60 ± 14	68 ± 17	63 ± 17	61 ± 15
NYHA III-IV, n [%]	5 (50%)	1 (10%)	13 (42%)	2 (6%)*
6-min walk distance [m]	–	540 ± 32	–	511 ± 93

LVESD, left ventricular end systolic diameter; EDV, end diastolic volume; ESV, end systolic volume; LA, left atrium; HR, heart rate; CO, cardiac output; CI, cardiac index; SV, stroke volume; LVP, left ventricular pressure; NYHA, New York Heart Association; NT-pro-BNP, N terminales pro brain natriuretic peptide; ECV, extracellular volume; aECV, absolute extracellular volume; GLS, global longitudinal strain; EF, ejection fraction;

^*^p < 0.05 not restored CircE pre-AVR vs not restored CircE post-AVR or restored CircE pre-AVR vs restored CircE post-AVR

^ +^ p < 0.05 not restored CircE pre-AVR vs restored CircE pre-AVR or not restored CircE post-AVR vs restored CircE post-AVR

### CircE in Aortic Stenosis

**Fig. 3:**
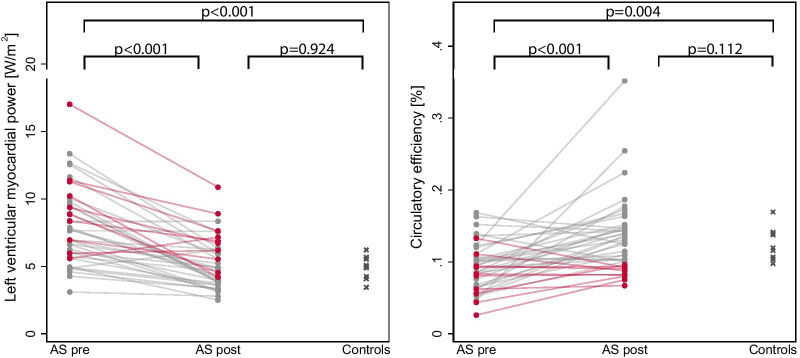
Left ventricular myocardial power (LVMP) and circulatory efficiency (CircE) in aortic stenosis (AS) before and after aortic valve replacement (AVR) and in controls

**Fig. 4:**
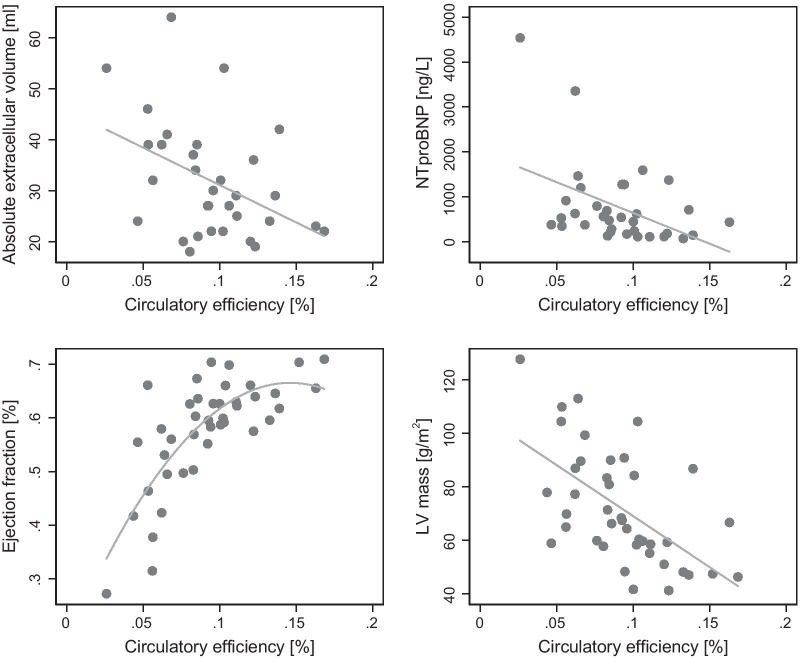
Correlation of circulatory efficiency (CircE) with absolute extra cellular volume (aECV), NTpro-BNP, LV ejection fraction and LV mass

**Fig. 5:**
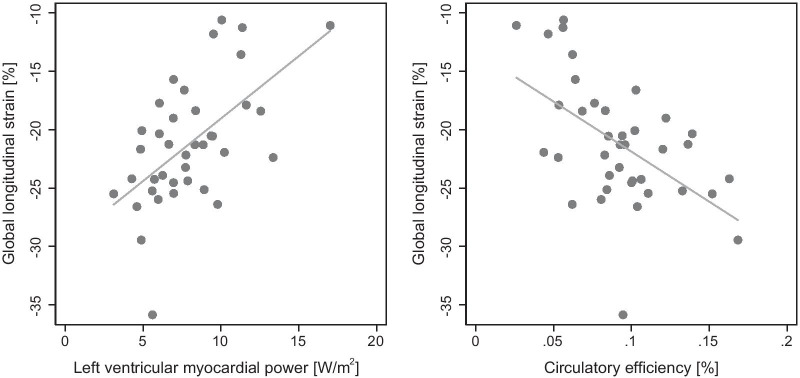
Correlation of LV myocardial power (LVMP) and circulatory efficiency (CircE) with global longitudinal strain (GLS)

In patients with severe AS, CircE was lower (9 ± 3 vs 12 ± 2%, p = 0.004) compared to healthy controls (Fig. 3). Furthermore, there were significant inverse correlations between pre-operative CircE and LV mass (r = − 0.591, p < 0.001), aECV (r = − 0.427, p = 0.015), ESV (r = − 0.609, p < 0.001), LVEF (r = 0.704, p < 0.001), NT-proBNP (r = − 0.444, p = 0.009) (Fig. 4) and GLS (r = − 0.539, p < 0.001) (Fig. 5).

LVMP was higher (8 ± 3 vs 5 ± 1 W/m^2^, p < 0.001) (Fig. 3) and CP was not different (1.3 ± 0.4 vs 1.1 ± 0.1 W, p = 0.097) compared to healthy controls. Pre-operative LVMP correlated significantly with GLS (r = 0.577, p < 0.001) (Fig. 5).

### CircE after aortic valve replacement

After AVR, CircE significantly increased in the total cohort (9 ± 3 vs 13 ± 5%, p < 0.001) and showed no difference to healthy controls (13 ± 5 vs 12 ± 2%, p = 0.112) (Fig. 3).

Furthermore, there were significant correlations between post-operative CircE and post-operative LV mass (r = − 0.409, p = 0.008), ESV (r = − 0.454, p = 0.003) and LVEF (r = 0.555, p < 0.001).

LVMP decreased after AVR (8 ± 3 vs 5 ± 2 W/m^2^, p < 0.001) and showed no differences to healthy controls (5 ± 2 vs 5 ± 1 W/m^2^, p = 0.924) (Fig. 3). CP was not changed after AVR (1.3 ± 0.4 vs 1.3 ± 0.4 W, p = 0.176) and showed no differences to healthy controls (p = 0.462).

There was no significant correlation between improvement in efficiency and symptom improvement (p = 0.721). Improvement of CircE significantly correlated to decrease of LVMP (R2 = 0.249, p = 0.001). Decrease of LVMP significantly correlated to changes of aortic pressure gradient (R2 = 0.321, p < 0.001), LVM (R2 = 0.451, p < 0.001), ESV (R2 = 0.243, p = 0.001), EDV (R2 = 0.110, p = 0.034) and LVEF (R2 = 0.180, p = 0.006). Furthermore, improvement of CircE did not correlate with prosthesis size (p = 0.409).

### CircE does not normalize in 24% of patients

The lowest value for CircE in controls was 10%. 10/41 (24%) patients displayed CircE of < 10% after AVR. Between the two groups without (n = 10) and with restored CircE (n = 31) after AVR, we found the following effects:

Pre-operative findings: In patients without restored CircE 70% were male, LVEF was lower (50 ± 13 vs 60 ± 8%, p = 0.031) and diastolic RR was higher (80 ± 6 vs 73 ± 11 mmHg, p = 0.015). There was no difference in CircE, mean gradient across the aortic valve, NYHA status or markers for hypertrophy or fibrosis (Tables 3 and 4). No parameter could be identified to predict which patient would show restored or not restored CircE after AVR.

Post-operative findings: Reduction in fibrosis (aECV) (32 ± 11 vs 26 ± 8 ml, p < 0.001), improvement in CircE (0.6 ± 2.8 vs 5.2 ± 6.0%, p = 0.009) and in NYHA (NYHA III-IV 42% vs 6%, p < 0.05) was only significant in patients with restored CircE. Improvement in LVEF (50% vs 57%, p < 0.05) was only significant in patients without restored CircE after AVR. Mean gradient across the aortic valve (14 ± 5 vs 10 ± 5 mmHg, p = 0.026) and LVMP (7 ± 2 vs 5 ± 2, p = 0.001) were higher in patients without restored CircE, however, there was no significant difference in post-operative LVP, NT-pro-BNP, cardiac function, NYHA status or markers for hypertrophy between patients with and without restored CircE. Furthermore, there were no differences in pre- to post-AVR changes of LV mass, aortic valve gradient and NT-proBNP between patients with and without restored CircE (ables 3 and 4).

### Patients with lower pre-operative CircE show a higher absolute amount of fibrosis after AVR

**Fig. 6:**
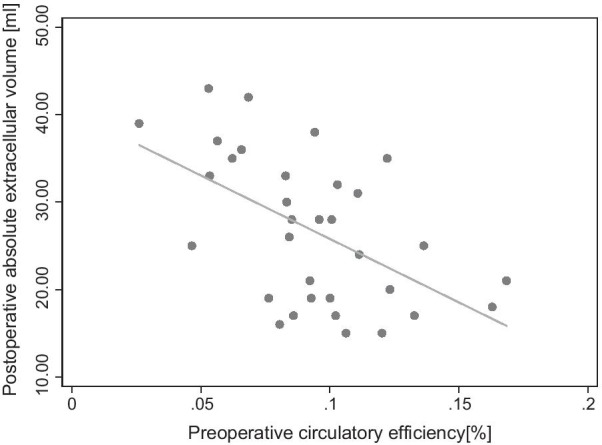
Correlation of preoperative circulatory efficiency (CircE) and postoperative absolute extra cellular volume (aECV)

Pre-operative CircE correlates inversely with aECV post-operative (r = − 0.542, p = 0.001) (Fig. 6).

## Discussion

We quantified a surrogate marker of circulatory efficiency (CircE) longitudinally in patients with severe AS before and after surgical AVR using a non-invasive CMR technique. We found CircE to be reduced in patients with AS and lower CircE was associated with pronounced LV hypertrophy and fibrosis and reduced LV function. After surgical AVR, CircE did not increase and normalize in an important fraction of patients (24%). These patients also showed less reduction in LV myocardial fibrosis volume compared to patients with restored CircE after AVR. Improvement of CircE was significantly influenced by the decrease of LVMP. Furthermore, decrease of LVMP was significantly affected by changes of aortic pressure gradient, LVM, ESV, EDV and LVEF. Therefore, decrease of LVMP and improvement of CircE is affected by the decrease of afterload but is also influenced by cardiac reverse remodeling.

Myocardial adaptation processes like hypertrophy and fibrosis in patients with AS lead to higher LV energy demand and reduced efficiency [1–3, 9]. If left untreated transition from adaptive to maladaptive remodeling can lead to heart failure [1, 2]. The concept of LVMP and efficiency as an evaluation of myocardial performance in pressure loaded hearts and in heart failure have increasingly become of interest [7, 11, 13, 14, 25, 26] since prior studies have demonstrated LV efficiency to be reduced in pressure overloaded hypertrophied hearts.

In hypertrophied and failing hearts, a switch from aerobic mitochondrial fatty acid oxidation to anaerobic glycolysis has been described, which decreases myocardial efficiency due to inefficient ATP generation and increased adenosine triphosphate (ATP) consumption for other non-contractile purposes [27]. Even in normal hearts 20% of O2 are consumed by biochemical processes not directly associated with contraction (e.g. electrolyte homeostasis) [28].

Myocardial efficiency is defined as the ratio between external work and myocardial energy consumption [9, 29]. The area of the pressure–volume loop reflects external work (stroke work) and can be measured by using invasive catheter. Myocardial oxygen consumption reflecting myocardial energy consumption has also been measured using invasive tools. This approach has become gold standard to measure myocardial energetics. However, this approach is limited by its invasive nature and therefore, has been limited to specific indications in clinical routine. Our approach quantifies surrogate markers of myocardial power and efficiency by using only non-invasive CMR-based volumetric and blood flow measurements. Hence, our approach can easily be applied in clinical routine and research. The advantage and motivation of the proposed surrogate markers were well discussed earlier [15]. As also shown in a prior study our approach reflects disease specific alterations of myocardial power and efficiency in hearts with chronic pressure- and volume overload [15].

In our study, we calculated circulatory efficiency by measuring mechanically generated power of the LV necessary to perform contraction against a given afterload following the law of Laplace and considering only geometrical parameters assessed by CMR. We recently demonstrated a reduced circulatory efficiency in patients with AS and different grades of severity [15]. The presented approach is merely noninvasive, however, not yet validated against invasive standards. In the present study, the focus was on patients with severe AS, who received AVR and calculated circulatory efficiency before and after AVR and we found similar results compared to study results using invasive methods.

Hansson and colleagues previously quantified efficiency in mainly asymptomatic AS patients with and without heart failure and demonstrated reduced efficiency in patients with impaired LVEF compared to controls [14]. Our findings are in line with such measurements, showing correlations between CircE and LVEF in patients with AS. Güclu and colleagues demonstrated reduced efficiency in AS patients compared to controls and described efficiency as an important determinant of functional improvement after AVR [13]. However, their study was limited by a small patient number (n = 10) and non-age-matched controls.

AVR has beneficial effects on prognosis mainly due to reverse remodeling [30, 31]. In this study, AVR reduced pressure load, LV hypertrophy and fibrosis as expected and improved NYHA status and LV function looking at the whole cohort. Furthermore, CircE increased after AVR and normalized in the majority of patients. Güclu and colleagues described increased efficiency after AVR without normalization [13]. However, their controls were not age-matched [32] and normal efficiency was described as 49%, which is inconsistent with prior studies quantifying efficiency (14–35%) [10, 14].

In our study, CircE did not normalize in 10 (24%) patients. Mean value for preoperative LVEF was lower and diastolic blood pressure was higher in the non restored group. However, looking at the individual 10 patients with non restored CircE after AVR, 4 patients displayed LVEF lower than 45%, but in 6 patients LVEF was higher than 56%, showing the heterogeneity of the non restored group. NT-proBNP did not reach statistical significance between restored and non restored group. However, in general, patients who did not restore after AVR seem to be the patient group with patients, who were slightly sicker, although not many significant differences could be found. Postoperatively, NYHA and aECV only improved in the restored group and LVEF only improved in the non-restored group. Mean aortic pressure gradient was higher in the non restored group, however, postoperative mean aortic pressure gradient of 15 mmHg does not seem to be clinically relevant. Furthermore, the combined parameter LV pressure, which is part of the formula of CircE, was not significantly different between groups.

Statistically, we did not find any preoperative parameter that was predictive for patients showing postoperative non restored CircE and we could also not describe a main component, which was causative for showing non restored CircE after AVR. Further studies are needed and the two groups, especially the non restored group, is too small, however, circulatory efficiency taking into account different risk factors (LV pressure, LV mass, LV geometry) might be useful to categorize patients with pressure overload, who have not yet surpassed cut off values of single parameters.

Only patients with restored CircE after AVR showed improvement of CircE and myocardial fibrosis after AVR. Similar results were demonstrated by Güclu and colleagues where 4 out of 10 AS patients without efficiency improvement after AVR did not improve in exercise capacity after AVR [13]. Hence, CircE may identify patients at risk for insufficient reverse remodeling and could thus help to optimize timing for intervention. In further studies circulatory efficiency could be calculated in patients with AS longitudinally over time to investigate relationship between circulatory efficiency and myocardial adaptations, onset of symptoms and the optimal timing for intervention. According to the present data we can only speculate.

We found high CircE to be associated with high GLS, which is a measure of subclinical LV dysfunction and a predictor of reverse remodeling and outcome after AVR [33–36]. GLS is promising for risk stratification in patients with AS and for finding the optimal time for treatment [33–36]. Correlation between GLS and CircE might suggest similar clinical relevance of Circ E for patients with AS. Current AS guidelines mainly respect aortic pressure gradient for clinical decision making and staging [16] although the external load is not associated to onset of symptoms and LV hypertrophy [30, 37].

Pressure overload can trigger cellular pathways that lead to myocardial adaptation processes such as hypertrophy and fibrosis and is associated with heart failure in the long term [1–3]. Interestingly, CircE is correlated to absolute fibrosis load before and after AVR and might be an important contributor for pathophysiological understanding of early adaptation processes.

In our cohort of patients with severe AS we describe a reduction of LV mass and absolute fibrosis volume after AVR, however, fibrosis fraction (ECV) increased short term after AVR. This is in line with longitudinal biopsy studies from 1989 and recent CMR studies from Treibel TA et al., who described different cohorts of patients with severe AS and AVR and postoperative faster regression of myocardial mass than regression of fibrosis, which leads to an initial increase of fibrosis fraction short term after AVR, but constant decrease of the absolute amount of fibrosis load [38, 39].

In regard to efficiency we found a correlation of low pre-operative CircE with high post-operative fibrosis load. Moreover, there is only a significant reduction in absolute fibrosis volume in patients with restored CircE after AVR and not in patients with non-restored CircE after AVR. This suggests that reduced CircE in patients with severe AS is accompanied with delay in reverse remodeling after AVR at least concerning diffuse fibrosis since regression of myocardial mass and normalization of EDV and ESV is seen in all patients. In line with this suggestion recent literature studied the impact of myocardial fibrosis in patients with AS on LV reverse remodeling after aortic valve therapy. It was described that higher amount of myocardial fibrosis pre-treatment was associated with delay in normalization of LV geometry and function but not per se with absence of reverse remodelling and clinical improvement after treatment [40].

There was a high prevalence of bicuspid aortic valve (BAV) patients in our cohort. Prior studies comparing severe AS in patients with BAV and trileaflet aortic valve have shown that patients with trileaflet AS have a greater prevalence of cardiovascular risk factors and worse survival after AVR [41]. However, in their study patients with BAV were less likely to have multiple comorbidities.

In the present study, we did not find differences in LV power and circulatory efficiency, nor in markers for hypertrophy or fibrosis between AS patients with BAV and trileaflet AS before and after AVR. Looking at the patient characteristic there were no differences in age, aortic pressure gradient and cardiovascular risk factors such as diabetes, arterial hypertension and dyslipidemia. BAV patients showed a lower systolic blood pressure (134 ± 3vs 147 ± 7 mmHg; p = 0.042) and lower pre-operative pulse pressure (59 ± 2 vs 75 ± 6; p = 0.012), however, this did not have a relevant impact on the other parameters. It might be, that BAV and trileaflet AS patients in our patient cohort, were more comparable in their patient characteristics than in other studies describing relevant differences between these patients.

In a former publication we have described abnormal flow profiles in the ascending aorta to be present before and after AVR in the majority of patients [42]. In other studies, abnormal flow profiles are described to be associated with increased viscous energy loss, which can be used as a measure of LV load [43]. Thus, abnormal flow profiles might additionally influence LV work load and circulatory efficiency. However, this was not part of the present study.

### Limitations

Computing of myocardial energetics focused on systole, since it accounts for the majority of the heart’s energy expenditure, without further consideration of the diastole, although diastolic relaxation is an active ATP-consuming process. However, little is known about myocardial energetics in diastole, and more research is needed to unveil the underlying mechanisms.

The parameter circulatory efficiency does not represent a true measurement but a mathematical formula that integrates the numerical information of a total of eight variables (i.e. myocardial wall volume). Because the parameter circulatory efficiency cannot be measured, neither as a single nor as a repeat measurement, intra- and/or inter-observer variabilities and scan-rescan variability cannot be computed. However, parameters of cardiac power and efficiency have been calculated using clinical established CMR LV volumetric and flow measurements. Good reproducibility of CMR LV volumetric, 2D and 4D flow measurements have been shown in several studies [22–24, 44, 45].

Moreover, this study was a purely mechanical approach without metabolic measurements of myocardial oxygen consumption derived by PET or invasive hemodynamic measurements that assumed LVMP to be the surrogate potential power generated by LV contraction following the simplified law of Laplace. Furthermore, the pressure recovery phenomenon was not considered since aortic pressure gradients were assessed using Doppler echocardiography as currently recommended by guidelines [16]. Future studies may help improve the method by using the continuity equation or model-based approaches. In addition, myocardial wall stress was calculated using a simplified approach to the law of Laplace. The geometrical shape of the LV as well as regional strain both determine myocardial wall stress and, subsequently, impact myocardial power. Therefore, more accurate models should be applied to calculate myocardial power more accurately in future projects.

## Conclusion

In summary, the quantification of a surrogate marker of CircE in patients with severe AS before and after AVR has been demonstrated using a non-invasive CMR-based approach.

CircE was reduced in patients with AS and lower CircE was associated with pronounced hypertrophy and fibrosis and reduced LV function. After AVR, CircE increased and normalized in the majority of patients. In 24% of patients, CircE did not normalize and these patients showed no improvement of myocardial fibrosis compared to patients with restored CircE after AVR.

CircE, reflecting a combined parameter of LV adaptation to increased workload, could be valuable in the search for finding optimal timing of intervention in patients with AS to improve optimal long-term outcomes.

## Data Availability

The datasets used and analysed during the current study are available from the corresponding author on reasonable request.

## Abbreviations

2D: Two-dimensional
4D: Four-dimensional
Σwall: Wall stress
AR: Aortic regurgitation
AS: Aortic stenosis
ATP: Adenosine triphosphate
AVR: Aortic valve replacement
BAV: Bicuspid aortic valve
BMI: Body mass index
BSA: Body surface area
CCS: Canadian Cardiovascular Society
CircE: Circulatory efficiency
CMR: Cardiovascular magnetic resonance
CO: Cardiac output
CO_eff_: Effective cardiac output
CP: Circulatory power
ECG: Electrocardiography
ECV: Extracellular volume
EDV: End diastolic volume
EF: Ejection fraction
ESV: End systolic volume
FA: Flip angle
FT: Feature tracking
GCS: Global circumferential strain
GLS: Global longitudinal strain
HR: Heart rate
LV: Left ventricle/left ventricular
LVEDV: Left ventricular end diastolic volume
LVESV: Left ventricular end systolic volume
LVM: Left ventricular mass
LVMP: Left ventricular myocardial power
MAP: Mean arterial pressure
NT-proBNP: N-terminal pro b-type natriuretic peptide
NYHA: New York Heart Association
PET: Positron emission tomography
PSYS: Peak systolic pressure
RBP: Mean radius of the blood pool
RF: Regurgitation fraction
SV: Stroke volume
S_Wall_: Mean myocardial wall thickness
t_ABC_: Auxobaric contraction time
TAV: Trileaflet aortic valve
t_CS_: Total systolic contraction time
t_IVC_: Isovolumetric contraction time
V_wall_: Myocardial wall volume
VENC: Velocity encoding
